# Uni-, Bi- or Trifocal Hepatocellular Carcinoma in Western Patients: Recurrence and Survival after Percutaneous Thermal Ablation

**DOI:** 10.3390/cancers13112700

**Published:** 2021-05-30

**Authors:** Ancelin Preel, Margaux Hermida, Carole Allimant, Eric Assenat, Chloé Guillot, Cecilia Gozzo, Serge Aho-Glele, Georges-Philippe Pageaux, Christophe Cassinotto, Boris Guiu

**Affiliations:** 1 Department of Radiology, St-Eloi University Hospital, 34980 Montpellier, France; a-preel@chu-montpellier.fr (A.P.); m-hermida@chu-montpellier.fr (M.H.); c-allimant@chu-montpellier.fr (C.A.); chloe-guillot@chu-montpellier.fr (C.G.); ceciliagozzo91@gmail.com (C.G.); c-cassinotto@chu-montpellier.fr (C.C.); 2 Department of Oncology, St-Eloi University Hospital, 34295 Montpellier, France; e-assenat@chu-montpellier.fr; 3 Department of Epidemiology & Biostatistics, Dijon University Hospital, 21000 Dijon, France; ludwig.aho@chu-dijon.fr; 4 Department of Hepatology, St-Eloi University Hospital, 34295 Montpellier, France; gp-pageaux@chu-montpellier.fr

**Keywords:** liver cancer, metastasis, prognosis, radiofrequency ablation, microwave ablation

## Abstract

**Simple Summary:**

Percutaneous thermal ablation (PTA) is a validated treatment for small (<3 cm) hepatocellular carcinoma (HCC). Multifocality is usually reported as a strong pejorative factor. Yet, the current literature lacks data on the influence in Western patients of HCC nodule numbers on recurrence and survival after PTA. From a prospective cohort of patients who underwent PTA for <3 cm HCC, we retrospectively compared recurrence and survival, according to the number of nodules. We found that bi- and trifocal HCC significantly increased the risk of distant recurrence, especially very early (<6 months) distant recurrence. Overall survival after PTA of trifocal HCC proved to be significantly below what was expected after a curative treatment, ranging between that of BCLC A and of BCLC B patients. Liver transplantation should certainly be considered earlier in this sub-population. Reasonable hopes come from adjuvant/neoadjuvant trials based on immunotherapies alone or in combination.

**Abstract:**

Multifocality is usually reported as a pejorative factor after percutaneous thermal ablation (PTA) of HCC but little is known in Western series. Recurrence and survival were extracted from a prospective database of all patients who underwent PTA for *≤*3 cm HCC. From January 2015 to April 2020, we analyzed 281 patients with unifocal (*n* = 216), bifocal (*n* = 46) and trifocal (*n* = 16) HCC. PTA of bi- and trifocal HCC resulted in a high risk of very early (<6 months) distant recurrence (38.8% and 50%, respectively). Median RFS was 23.3 months (95% CI:18.6–30.4), 7.7 months (95% CI:5.1–11.43, *p* = 0.002) and 5.2 months (95% CI:3–12.3, *p* = 0.015), respectively, for uni-, bi- and trifocal HCC groups. In a multivariate analysis, both bifocal (HR = 2.46, *p* < 0.001) and trifocal (HR = 2.70, *p* = 0.021) vs. unifocal HCC independently predicted shorter RFS. Median OS in trifocal HCC group was 30.3 months (95 CI:19.3-not reached). Trifocal vs. unifocal HCC independently predicted shorter OS (HR = 3.30, *p* = 0.008), whereas bifocal vs. unifocal HCC did not (*p* = 0.27). Naïve patient (HR = 0.42, *p* = 0.007), AFP > 100 ng/mL (HR = 3.03, *p* = 0.008), MELD > 9 (HR = 2.84, *p* = 0.001) and steatotic HCC (HR = 0.12, *p* = 0.038) were also independent predictors of OS. In conclusion, multifocal HCCs in a Western population have a dramatically increased risk of distant recurrence. OS after PTA of trifocal HCC is significantly below what was expected after a curative treatment.

## 1. Introduction

Hepatocellular carcinoma (HCC) is the fifth most common cancer and the second most frequent cause of cancer-related deaths globally [1,2]. HCC-incidence, which is expected to increase in the future due to the aging of the population and to the emergence of non-alcoholic fatty liver disease (NAFLD) [3], is a major global health problem.

Percutaneous thermal ablation (PTA) is a validated first-line treatment for very early and early HCC, corresponding to Barcelona Clinic Liver Cancer (BCLC) 0/A patients, i.e., up to trifocal HCC < 3 cm, in patients with good health status (Eastern Cooperative Oncology Group ECOG−0) and well-preserved liver function (Child-Pugh A class) [1,4].

Outcomes after PTA were extensively investigated in Asian cohorts, i.e., in a context of predominant viral-induced liver disease. In these cohorts, PTA was mainly performed using ultrasonography (US) guidance alone, leading to the selection of only those patients with HCC both visible and accessible under US. As after resection, a high rate of five-year cumulative distant recurrence was reported (58 to 81%) [5,6,7,8,9,10]. Even though tumor characteristics (size, multifocality, serum α-fetoprotein (AFP) level) were shown as strong predictors of recurrence and survival [5,6,9,10,11,12] in this HCC population, little is known in Western series where the number of HCC nodules has only been studied as a binary variable (uni- vs. multifocal) [6,7,9,12].

The American Association for the Study of Liver Disease (AASLD) and the European Association for the Study of the Liver (EASL) guidelines clearly indicate that PTA is recommended as first-line therapy for very early solitary HCC (BCLC 0), even in surgical candidates [1]. However, these guidelines remain more elusive in cases of small multifocal HCCs. Therefore, the results of PTA in BCLC 0-A multifocal HCCs are worth exploring in Western patients, especially in a context where PTA is applied without any selection bias regarding US visibility/accessibility.

The aim of this study was to explore recurrence and survival after PTA of small HCCs in Western patients, according to the number of tumor nodules.

## 2. Materials and Methods

### 2.1. Design and Ethics

We conducted a retrospective study based on a prospective database of all the patients who underwent PTA for HCC at our institution. This study was approved on 22 April 2021 by our institutional review board (ClinicalTrial.gox Identifier: NCT03428321 ) and written informed consent for both the procedure and the prospective anonymized data collection was obtained from all patients.

### 2.2. Patients and Tumor Data

Inclusion criteria were: (a) adults undergoing PTA for 1–3 nodules of HCC diagnosed by either biopsy or imaging criteria according to EASL/AASLD guidelines (only LI-RADS 5 nodules were considered) [1,4]. Treatment by PTA was decided upon during our bi-weekly multidisciplinary meeting on liver tumors, which gathered interventional radiologists, liver surgeons, oncologists, hepatologists and radiation oncologists. PTA was considered a first-line option according to Barcelona Clinic Liver Cancer (BCLC) criteria for BCLC 0-A patients [3]; (b) Child-Pugh score <B8; (c) World Health Organization performance status 0 or 1; (d) prothrombin time ratio >50%, (e) and platelet count higher than 50 G/L.

Exclusion criteria were: HCC > 30 mm, follow-up <3 months, peri-hilar tumor (of note, this criterion was our sole technical contraindication to PTA), history of biliary-digestive anastomosis or endoscopic sphincterotomy; combined treatment with embolization or chemoembolization.

The following patient and liver characteristics were collected: age, sex, body mass index (BMI), diabetes mellitus, cirrhosis (defined as typical hepatic dysmorphia on imaging, or by non-invasive evaluation of fibrosis, or by histological analysis of liver biopsy; patients were considered noncirrhotic based on liver biopsy or non-invasive evaluation of fibrosis; the others were considered undetermined) and cause of cirrhosis, biological markers (including alpha-fetoprotein (AFP) level), Child-Pugh and MELD scores, Albumin–bilirubin (ALBI) score. All patients underwent baseline liver magnetic resonance (MR) scans. The presence of steatosis and the amount of liver fat content was assessed on chemical-shift gradient-echo imaging [13]. The anesthesiologist evaluated the American Society of Anesthesiologists physical status score (ASA). Tumor characteristics were also noted: number of HCC nodule(s), size of largest lesion, and the steatotic or non-steatotic nature of HCC (defined as signal intensity loss on opposed-phase compared with in-phase gradient echo images for at least one HCC nodule). Dome (defined when the lung parenchyma was interposed between the skin and the tumor through the anterior or lateral route on the axial plane on baseline imaging), subcapsular location (i.e., direct contact with the liver capsule) and peri-vascular tumor (i.e., located ≤5 mm from any liver vessel) were also recorded [12].

### 2.3. Percutaneous Thermal Ablation

All procedures were performed under general anesthesia in a multimodality interventional suite incorporating an Angio-CT system (Infinix-I 4 DCT, Canon Medical Systems, Tokyo, Japan) and an ultrasound machine (Logiq E9, General Electric, Milwaukee, WI, USA). PTA was performed by four interventional radiologists (5–15 years of expertise in liver PTA) using radiofrequency or microwave device, depending on the operator’s choice. It was intended to have a 5–10 mm ablation margin all around the tumor. Ultrasonography (US) was the first-line guidance modality. Our approach was multimodal [12] so that no case was excluded for technical reasons (tumor location, visibility, etc.). When the tumor was not visible or accessible under US, we used intra-arterial tumor tagging by ethiodized oil (Lipiodol, Guerbet, France) and CT guidance. In addition, techniques such as carbo-dissection or hydro-dissection were used whenever necessary to prevent collateral damage on at-risk anatomical structures closed to the tumor(s) [12]. Contrast-enhanced CT (portal phase) was performed immediately after the procedure both to evaluate the ablation zone (i.e., the area of low attenuation) and to detect post procedural complications. Patients were discharged on day 1, except when a complication occurred.

### 2.4. Follow-Up and Outcomes

Complications were recorded according to Society of Interventional Radiology (SIR) guidelines [14]. SIR-A complications considered as minor and/or subjective without any consequence were not routinely collected.

Clinical, biological evaluations (including AFP level and liver function test), and imaging follow-up were performed 6 weeks after PTA and then every 3 months. Imaging included contrast-enhanced MRI (except in very rare cases of incompatible pacemaker or claustrophobia, for whom quadriphasic contrast-enhanced CT was conducted instead) and a chest CT-scan every 6 months.

Local tumor progression (LTP) was defined as any growing or enhanced tumor focus within, or at the edge (direct contact) of, the ablation zone, after complete ablation documented by at least one MRI. Distant recurrence was the emergence of one or several new HCCs not adjacent to the ablation zone or of any extra-hepatic recurrence (diagnosed by either biopsy or imaging follow-up).

For each patient, the first three occurrences of both LTP and distant recurrences were recorded. For each recurrence, tumor number, size of largest lesion, vascular invasion, the presence of extra-hepatic metastases and AFP serum level were systematically collected.

### 2.5. Statistical Analysis 

The continuous variables were described using means ± standard deviation (SD) for those normally distributed and medians and interquartile range (IQR) for those not normally distributed. Variables were compared with the Fischer’s exact test or the Kruskal–Wallis test, as appropriate.

Time-to-distant recurrence was defined from PTA to the first distant recurrence. Recurrence-free survival (RFS) was defined as the time from PTA to the first recurrence, death or to last follow-up. Overall survival (OS) was defined as the time from PTA to death (all causes). Median follow-up with its 95% CI was calculated using the reverse Kaplan–Meier method. Covariates associated with OS, RFS and time-to-distant recurrence were explored using Cox proportional-hazards models. For all time-to-event analyses (i.e., OS, RFS, time-to-distant recurrence), patients who underwent liver transplantation were censored at transplantation date to take into account this competing risk. Multivariate Cox models for RFS and OS were constructed by including significant variables by univariate analysis. A robust variance estimator was used systematically. Log-linearity was checked using fractional polynomials.

We also analyzed covariates associated with the occurrence of very early (i.e., <6 months) distant recurrence by using a logistic regression model. For each multivariate Cox model, we evaluated the Akaike information criterion (AIC) for goodness of fit and Harrell’s C-statistic for discrimination (where no predictive discrimination would have a Harrell’s C index of 0.5 and perfect separation of patients, a Harrell’s C index of 1.0). For multivariate logistic regression models, the area under the ROC (AUROC) curve was computed to capture the predicting ability of the model. All multivariate models were internally validated using bootstrapping (200 replications).

All analyses were performed with the Stata software, version 17 (Stata corporation, College Station, TX, USA). A *p*-value < 0.05 was considered significant.

## 3. Results

### 3.1. Patient Characteristics

**Figure 1:**
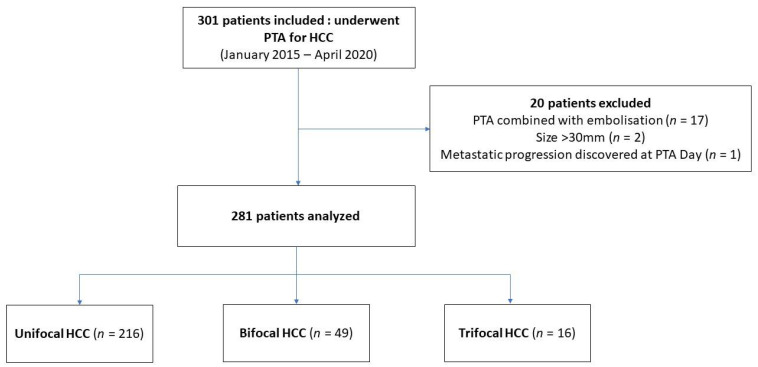
Flowchart.

From January 2015 to April 2020, we included 301 patients who underwent PTA for small HCC (Figure 1). Twenty patients were excluded due to: combined treatment with transarterial (chemo)embolization (TACE) (*n* = 17), tumor size > 30 mm (*n* = 2), metastatic progression discovered at PTA day (*n* = 1). Finally, we analyzed 281 patients, among whom 216 had unifocal HCC, 46, bifocal and 16, trifocal.

Baseline characteristics of patient, liver, tumor(s), and PTA technique are collected in Table 1 and did not differ between uni-, bi- and trifocal HCCs, except with respect to the presence of steatotic HCC (more frequent in unifocal HCC) and perivascular tumors (more frequent in unifocal HCC). Globally, the median age was 65 years (IQR 59–72) and 225/281 (80.1%) were men. Nearly half (138, 49.1%) were HCC-naïve. Alcohol abuse was noted in 56.5% of patients (either alone or in addition to viral hepatitis). Cirrhosis was detected in 258 patients (91.8%). Nevertheless, liver function was well-preserved (97.5% were Child-Pugh A, median MELD score was 8 (IQR 7–10)). Radiofrequency ablation was used in 122 patients (43.4%), and microwave ablation was used in 159 patients (56.6%). Guidance imaging modality was ultrasonography in 52.3% of cases.

### 3.2. Follow-Up and Events

Remarkably, no patient was lost to follow-up over the study period. The median follow-up in the whole cohort was 36.6 months (95% CI: 33.0–41.0) and this did not differ between uni-, bi-, and trifocal HCC groups. Complications (SIR B-E) occurred in 17 patients (6.05%), without a significant difference between groups. No PTA-related death, needle track seeding, or liver abscess was reported. Fifty-four patients (19.2%) presented LTP during follow-up (18.5% in unifocal, 26.5% in bifocal and 6.3% in trifocal HCC), but LTP occurrence did not differ between the three groups (*p* = 0.129). During follow-up, 31 patients (11%) underwent liver transplantation and 72 patients (25.6%) died.

### 3.3. Distant Recurrence

During follow-up, 145 patients (51.6%) developed at least one distant recurrence. Distant recurrence occurred in 94 patients (43.5%) with unifocal, 37 patients (75.5%) with bifocal and 14 patients (87.5%) with trifocal HCC (*p* < 0.001).

The 6-month, 1-, 2- and 3-year cumulative distant recurrence rates were 17.9% (95% CI: 13.9–22.1%), 32% (95% CI: 26.8–38%), 47.8% (95% CI: 41.6–54.4%) and 57.7% (95% CI: 50.7–64.9%), respectively, in the whole cohort.

The 6-month, 1-, 2- and 3-year cumulative distant recurrence rates for the uni-, bi- and trifocal groups are listed in Table 2.

**Figure 2:**
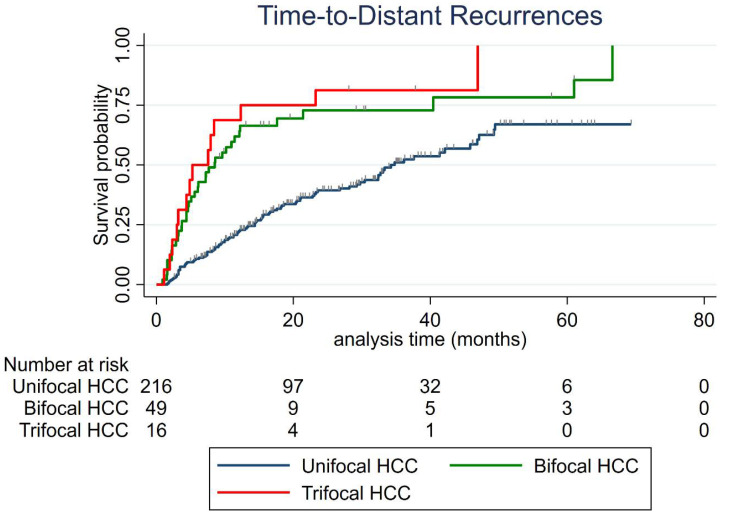
Time-to-distant-recurrence according to the number of HCC nodule (s).

Median time-to-distant-recurrence for the whole cohort was 29.8 months (95% CI: 21–36.2). Median time-to-distant-recurrence was 34.3 months (95% CI: 29.8–46.9) for unifocal HCC, 8.5 months (95% CI: 5.1–12.1) for bifocal HCC (vs. unifocal, *p* < 0.001) and 5.3 months (95 CI%: 3–12.3) for trifocal HCC (vs. unifocal, *p* < 0.001), respectively (Figure 2).

Variables independently associated with the occurrence of very early (<6 months) distant recurrence in multivariate logistic regression analysis were bifocal vs. unifocal (OR = 5.16, *p* < 0.001), trifocal vs. unifocal HCC (OR = 7.18, *p* = 0.002), AFP > 100 ng/mL (OR = 16.6, *p* < 0.001) and HCC-naïve patient (OR = 0.26, *p* = 0.001). These results were internally validated using bootstrapping. In addition, the predicting ability of the multivariate model was high (AUROC = 0.8).

The patterns of the first distant recurrence were examined in Table 3.

The number of HCC nodule(s), their maximal diameter, AFP serum level, and the occurrence of portal vein invasion or extra-hepatic metastasis did not differ between groups. Globally, no curative treatment could be applied in 40.4% of cases, without any difference between groups.

### 3.4. Recurrence-Free Survival (RFS)

**Figure 3:**
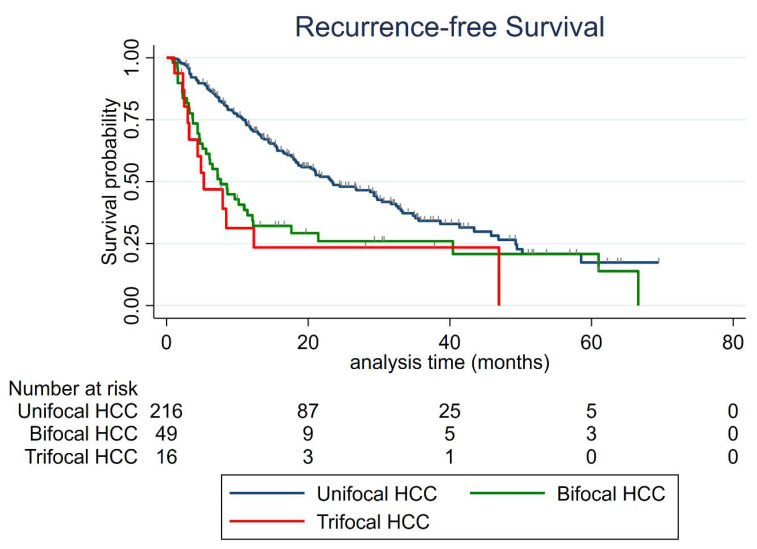
Recurrence-free survival according to the number of HCC nodules.

As depicted in Figure 3, the median RFS of the whole cohort was 18.6 months (95% CI: 15.6–23.6). The median RFS was 23.3 months (95% CI: 18.6–30.4) for the unifocal HCC group; 7.7 months (95% CI: 5.1–11.43) for the bifocal HCC group (vs. unifocal *p* = 0.002); and 5.2 months (95% CI: 3–12.3) for the trifocal HCC group (vs. unifocal *p* = 0.015). 

Factors associated with RFS in univariate analysis are listed in Table 4. In multivariate analysis, bifocal vs. unifocal (HR = 2.46, *p* < 0.001), trifocal vs. unifocal HCC (HR = 2.70, *p* = 0.021), HCC-naïve patient (HR = 0.46, *p* < 0.001) and AFP > 100 ng/mL (HR = 3.31, *p* < 0.001) independently predicted shorter RFS. These results were internally validated using bootstrapping.

### 3.5. Overall Survival (OS)

During follow-up, 52 patients (24.1%) with unifocal, 11 patients (22.5%) with bifocal and nine patients (56.3%) with trifocal HCC died (*p* = 0.025). Death was related to HCC progression in 22/52 (42.3%) unifocal, 9/11 (81.8%) bifocal and 8/9 (88.9%) with trifocal HCC patients (*p* = 0.005).

The 1-, 2- and 3-year overall survival rates were 96.9% (95% CI: 93.9–98.5%), 83.9% (95% CI: 78.3–88.2%) and 74.7% (95% CI: 67.7–80.4), respectively, in the whole series.

The 1-, 2- and 3-year overall survival rates for each group of uni-, bi- and trifocal HCC patients are listed in Table 5.

**Figure 4:**
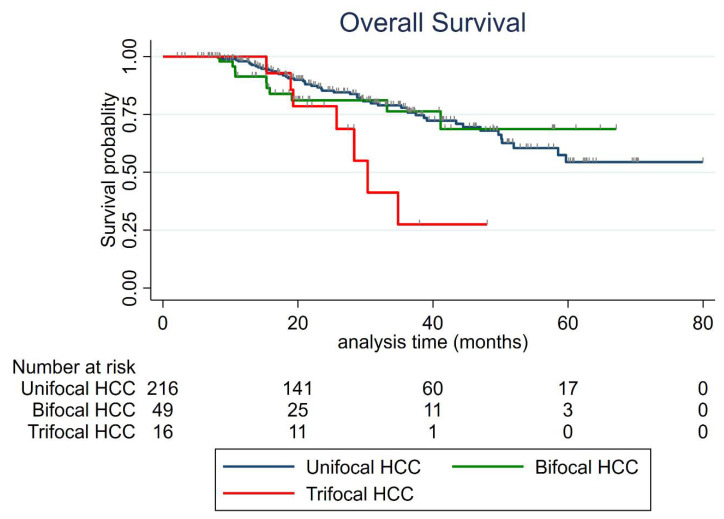
Overall survival according to the number of HCC nodules.

The median OS was not reached for all groups, except for trifocal HCCs (30.3 months (95 CI:19.3-not reached)) (Figure 4). Factors associated with OS in uni- and multivariate analysis are listed in Table 6. Trifocal vs. unifocal HCC was independently associated with shorter OS (HR = 3.30 (95% CI: 1.37–8.02) *p* = 0.008), whereas bifocal vs. unifocal HCC was not (HR = 1.60 (95% CI: 0.69–3.72) *p* = 0.27). Naïve patient (HR = 0.42, *p* = 0.007), AFP > 100 ng/mL (HR = 3.03, *p* = 0.008), MELD > 9 (HR = 2.84, *p* = 0.001) and steatotic HCC (HR = 0.12, *p* = 0.038) were also found as independent predictors of overall survival. The predicting ability of the multivariate model was high (Harrel’s C index = 0.79). The results were internally validated using bootstrapping, except for those related to steatotic HCC.

## 4. Discussion

Although PTA is a clearly validated treatment of BCLC 0-A HCC, data are lacking in the literature with respect to the impact on the outcome of the number of HCC nodules in Western patients, who mainly present non-viral liver disease. The occurrence of distant recurrence was three times higher in bi- and trifocal versus unifocal (the 1-year cumulative distant recurrence rates were 21.8%, 61.9% and 68.8%, for uni-, bi-, and trifocal HCC, respectively). PTA of bi- and trifocal HCC resulted in a high risk of very early (<6 months) distant recurrence (38.8% and 50%, respectively). In a context of curative treatment, trifocal HCC patients exhibited short OS (27.5% at 3 years), compared with the OS reported for uni- and bifocal HCC groups. 

Most cohorts [5,10,15,16,17,18,19,20,21] reporting on PTA for early-stage HCC come from Asia, where viral cirrhosis represents around 80% of liver disease, by contrast with the Western population, whose cirrhosis is mainly due to alcohol (57% of alcohol-induced liver disease, 25% of viral cirrhosis and 15% of NASH in our series). Higher mortality and recurrence rates are observed in patients with alcohol-related HCC, compared to viral etiology [22]. Among Asian studies, only a few [16,18,19,23] described the outcomes after PTA according to the number of HCC nodules. In a large Japanese cohort study, Shiina et al. [6] revealed that multifocal HCC was significantly related to distant recurrence (HR = 1.36 (95% CI: 1.16–1.59)) and shorter survival (the 5-year overall survival was 54.4% for multifocal HCC, compared to 64.6% for unifocal), but their analysis did not discriminate between bi- and trifocal HCC. Moreover, in most studies [5,6,7,8,9,10,21], PTA was performed under ultrasonography (US) guidance alone, whereas 45–54% of HCC candidates to PTA are invisible or inconspicuous under US [10,24], thereby leading to considerable selection bias. Here, we used a multimodal strategy (ultrasonography, CT scan with or without tumor tagging) to address all small HCC cases, and only considered central location as a technical contraindication. This strategy is likely to become the reference method [12,25]. In the literature, there is great debate regarding the best treatment option between PTA and surgical resection in early-stage HCC [1,26] in the absence or in case of limited portal hypertension. Obviously, high LTP rates after PTA do translate into lower RFS [27], but this must be toned down by the fact that LTP remains a relatively rare event (<25%), accessible to new PTA session in >80% cases [9]. The issue of distant recurrence is shared by both surgical resection and PTA.

In our series, time-to-distant-recurrence strongly differed between uni-, bi- and trifocal HCC groups (34.3 months, 8.5 months and 5.3 months, respectively). Not surprisingly, this translated into significant differences in RFS (23.3 months, 7.7 months, and 5.2 months, respectively). In 2019, Sempokuya and Wong [28] demonstrated that HCC patients who survived over 10 years after a curative treatment (liver transplantation, resection or ablation) had a mean time to first recurrence of 57.1 months, versus 15.3 months for those who did not survive. Classically, early HCC relapse (within 2 years) is considered as a metastatic phenomenon, whereas late relapse rather represents de novo carcinogenesis [29]. Tabrizian P et al. [30] reported that a time from resection to first HCC recurrence <1 year was associated with shorter survival (in 356 patients who developed recurrence, the median survival was 14.3 months for those who developed recurrence before 1-year post-resection, versus 28.7 months for those who developed recurrence 1–2 years after resection; *p* < 0.001). Tabrizian P et al. chose a 1-year cut-off because it was previously used in other cancers such as colorectal liver metastases [31,32]. A recurrence of one year, which can certainly be considered as early recurrence, frequently occurred in multifocal HCCs in our series (61.9% and 68.8% in bi- and trifocal HCC, respectively). We aimed to apply a more stringent approach by looking at very early (<6 months) distant recurrence. In the trifocal HCC group, 50% of patients had already relapsed at 6 months after PTA (compared to 11% and 39% for uni- and bifocal HCC, respectively), certainly revealing a dramatic rate of occult (i.e., radiologically-undetected) metastases. In 2018, based on 3696 patients from the United States who underwent liver transplantation, Aufhauser et al. [33] reported a 37% rate with occult metastasis on explant pathology, which is similar to their 2-year distant recurrence rate, thereby suggesting that occult metastasis from primary HCC was the main cause of early recurrence after resection. These observations call for a strict follow-up after PTA. Moreover, radiologists should be aware of the risk of very early recurrence in bifocal HCC, which is even higher in trifocal HCC.

We made the assumption that the earlier the tumor recurs, the earlier the patient is likely to drift to palliative treatment (≈40% in our series at first distant recurrence) and therefore, to worsen their prognosis. This might explain the median OS observed in trifocal HCC (30.3 months), which can be regarded as short for a curative treatment. Indeed, according to AASLD and EASL recommendations [34,35], median OS should be over 6 years in early HCC stage, but over 26–30 months in intermediate HCC stage. Interestingly, Zhang et al. [18] reported (in an Asian cohort) 5-year OS rates of 50.9% in the bifocal subgroup versus 17.5% in the trifocal subgroup (*p* = 0.001) in small multifocal HCC treated either by percutaneous, laparoscopic or by open surgical thermal ablation. Thus, the prognostic profile of small trifocal HCC, basically classified as BCLC A, is actually closer to that of BCLC B patients. In terms of prognosis profile, small trifocal HCC might be restaged in-between BCLC A and B, thereby reinforcing the necessity for BCLC subclassification in BCLC A patients, as was recently carried out in BCLC B/C patients [36,37]. These findings could also raise the question of treating these patients upfront with transarterial chemoembolization (TACE) rather than PTA. In a Japanese cohort, Takayasu et al. [38] reported a median survival of 37 months after TACE for trifocal HCC < 3 cm. Again, the context of predominant viral underlying liver disease is very different from ours in Western populations. In addition, TACE carries a 24.4–48% risk [39,40] of hepatic arterial damage, which may further impair intra-arterial therapies. A reasonable option, which may ultimately improve OS, would consist of treating small multifocal HCCs with repeated PTAs for as long a period as possible, in order to keep arteries patent, and to preserve the possibility of further intra-arterial treatments. However, this approach was actually that used in most cases in our series. Our results clearly show that there is room for improvement. One solution would be to consider liver transplantation earlier, especially in trifocal HCC patients. The dramatic rate of very early recurrence strongly urges the need to put the patient on the waiting-list from the onset, and to use PTA as a bridge to transplant. According to our results, salvage transplantation might be preferred in bifocal HCC patients, even though waiting for the first tumor relapse incurs the risk of facing non-transplantable recurrence. The optimal strategy is yet to be determined in the well-known context of graft shortage.

Another strategy for improving the prognosis of these patients relates to adjuvant and/or neoadjuvant treatments. Very recently, the landscape of advanced-HCC treatment has changed with the combination of atezolizumab (anti PD-L1) and bevacizumab (anti-VEGF), which has become the standard treatment [41]. There is a strong rationale for combining PTA with immune checkpoint inhibitors, in order to drive anti-tumor immune response [42,43,44,45]. Such an approach makes sense, as it limits the risk of distant relapse after PTA, and therefore the risk of ending up in palliative care. Several neoadjuvant or adjuvant trials are ongoing. Our team is conducting a multicenter randomized trial evaluating RFA of small HCC in combination with adjuvant atezolizumab and neoadjuvant atezolizumab/bevacizumab (AB-LATE02 trial, ClinicalTrials.dov Identifier: NCT04727307), with a stratification on the number of HCC nodules, in keeping with the results of this series. Finally, we confirmed here in a larger series with longer follow-up that steatotic HCC was independently associated with improved OS [46]. Steatotic HCC tumors belong to the G4 molecular sub-class with more favorable prognosis and less frequent multifocality [47], as also noticed in this study.

Several limitations to our study must be acknowledged. Firstly, it is a retrospective and monocentric study. However, we conducted a strict clinical, biological and imaging follow-up over a long time (median: 36.6 months) with no patient lost to follow-up, and had our analyses internally validated using bootstrapping. Secondly, we chose to include HCC-naïve and non-naïve patients, which might be regarded as a confounding bias. However, we adjusted for this covariate in our analyses. HCC-naivety was actually independently associated with the occurrence of very early recurrence, RFS and OS, thereby highlighting that a patient presenting a very first HCC had a very different disease course and prognosis from patients with a past history of HCC. Given its high significance, we also stratified on this factor in the AB-LATE 02 randomized trial. Thirdly, a relatively low number of patients underwent liver transplantation during follow-up, due to allocation rules in France and graft shortage. Finally, as often in PTA studies, we performed a very limited number of tumor biopsies, which precluded us from investigating the influence of pathological subtypes of HCC.

## 5. Conclusions

In conclusion, we showed that bi- and trifocal HCCs in a Western population have a dramatically increased risk of distant recurrence with a very early median time-to-recurrence (close to 6 months after PTA), which translates into poorer RFS. OS after PTA of trifocal HCC proved to be significantly below what was expected after a curative treatment, ranging between that of BCLC A and of BCLC B patients. Liver transplantation should certainly be considered earlier in this sub-population. Reasonable hopes come from adjuvant/neoadjuvant trials based on immunotherapies alone or in combination.

## Figures and Tables

**Table 1:** Baseline characteristics of patients with HCC treated by PTA.

**Variables**	**Global**	**Unifocal**	**Bifocal**	**Trifocal**	***p*** **-Value**
Patients	281	216	49	16	
Age (median (IQR) years)	65 (59–72)	66 (60–73)	63 (57–69)	58.5 (52.5–65.5)	0.082
Sex (*n*, %)					
Male	225 (80.07)	170 (78.70)	42 (85.71)	13 (81.25)	0.568
Female	56 (19.93)	46 (21.30)	7 (14.29)	3 (18.75)	
ASA score (*n*, %)					0.253
1–2	146 (51.96)	110 (50.92)	30 (61.22)	6 (37.50)	
3–4	135 (48.04)	106 (49.08)	19 (38.78)	10 (62.50)	
Diabetes (*n*, %)					0.534
No	170 (60.50)	124 (57.41)	33 (67.35)	13 (81.25)	
Yes	111 (39.50)	92 (42.59)	16 (32.65)	3 (18.75)	
Metformin treatment (*n*, %)	52 (18.51)	43 (19.91)	7 (14.29)	2 (12.50)	0.627
Statin treatment (*n*, %)	49 (17.44)	40 (18.52)	8 (16.33)	1 (6.25)	0.648
BMI (median (IQR) kg/m^2^)	27 (24–30)	27 (24–30)	27 (24–31)	27 (24–29.5)	0.399
Prior treatment for HCC (*n*, %)					0.264
Naïve patient	138 (49.11)	106 (49.07)	27 (55.10)	5 (31.25)	
Yes	143 (50.89)	110 (50.93)	22 (44.90)	11 (68.75)	
PTA (in medical history)	58 (20.64)	49 (22.69)	6 (12.24)	3 (18.75)	
Liver disease					
Cirrhosis (*n*, %)					1.000
No	23 (8.19)	18 (8.33)	4 (8.16)	1 (6.25)	
Yes	258 (91.81)	198 (91.67)	45 (91.84)	15 (93.75)	
Causes for hepatopathy (*n*, %)					0.23
Alcohol	115 (40.9)	83 (38.4)	25 (51)	7 (43.8)	
Viral hepatitis or mixed	113 (40.2)	88 (40.7)	19 (38.8)	6 (37.5)	
NASH	41 (14.6)	36 (16.7)	2 (4.1)	3 (18.7)	
Hemochromatosis and others	12 (4.3)	9 (4.2)	3 (6.1)	0 (0)	
Steatosis (*n*, %)					0.430
Absent	176 (64.23)	138 (65.71)	30 (62.50)	8 (50.00)	
Present	98 (35.77)	72 (34.29)	18 (37.50)	8 (50.00)	
MR quantification (median (IQR) %)	3 (2–6)	3 (2–6)	3 (2–6)	5 (3–10)	0.547
Child-Pugh class					0.511
A5	235 (83.6)	183 (84.7)	39 (79.6)	13 (81.2)	
A6	39 (13.9)	27 (12.5)	9 (18.4)	3 (18.8)	
B7	7 (2.5)	7 (3.2)	0 (0.0)	0 (0.0)	
MELD score (median (IQR))	8 (7–10)	8 (7–10)	10 (7–13)	8.5 (8–10)	0.144
MELD score > 9	95 (33.81)	68 (31.48)	20 (40.82)	7 (43.75)	0.300
Laboratory data (median (IQR))					
AFP (ng/mL)	5.2 (7.7)	4.7 (6.3)	7.6 (20.8)	7.7 (12.4)	0.094
Total bilirubin (µmol/l)	11 (10.2)	11 (9.6)	12 (9)	12 (13.1)	0.368
Albumin (g/l)	41 (6)	41 (6)	40 (6)	41.5 (6.5)	0.707
Prothrombin activity (%)	85 (23)	86 (22)	78 (23)	84 (23.5)	0.102
Platelet count (×10/mm^3^)	124 (91)	132 (96)	100.5 (116.5)	100.5 (116.5)	0.231
Platelet count ≤ 90000/ mm**^3^** (*n*, %)	96 (34.16)	66 (30.56)	22 (44.90)	8 (50.00)	0.063
Neutrophiles (×10/mm^3^)	3.28 (1.63)	3.32 (1.59)	3.16 (1.85)	3.65 (2.51)	0.813
Lymphocytes (×10/mm^3^)	1.42 (0.91)	1.41 (0.92)	1.42 (0.95)	1.65 (1.19)	0.879
Monocytes (×10/mm^3^)	0.51 (0.27)	0.52 (0.28)	0.49 (0.22)	0.56 (0.24)	0.812
Creatinine (µmol/L)	75 (28)	76.5 (29)	71 (18.40)	66.5 (26)	0.057
ALBI score					0.839
1	179 (66.54)	139 (67.48)	30 (63.83)	10 (62.50)	
2	90 (33.46)	67 (32.52)	17 (36.17)	6 (37.50)	
HCC					
Size of the largest nodule (median (IQR) mm)	16 (13–20)	15 (12–20)	17 (13–20)	17 (16–21)	0.099
Tumor size < 20 mm (*n*, %)	197 (70.11)	154 (71.30)	32 (65.31)	11 (68.75)	0.696
At least one biospy-proven nodule (*n*, %)	55 (19.6)	42 (19.4)	10 (20.4)	3 (18.8)	0.96
Subcaspular location (*n*, %)	105 (37.4)	77 (35.7)	23 (46.9)	5 (31.3)	0.31
Dome location (*n*, %)	70 (24.9)	60 (27.8)	7 (14.3)	3 (18.8)	0.11
Peri-vascular tumor (*n*, %)	64 (22.8)	58 (26.8)	5 (10.2)	1 (6.3)	0.01
Steatotic HCC (*n*, %)	57 (22.27)	51 (25.63)	4 (9.30)	2 (14.29)	0.046
PTA					
PTA modality *(n*, %)					0.857
Radiofrequency	122 (43.42)	92 (42.59)	23 (46.94)	7 (43.75)	
Microwave	159 (56.58)	124 (57.41)	26 (53.06)	9 (56.25)	
Imaging guidance (*n*, %)					0.369
Ultrasonography guidance	147 (52.31)	119 (55.09)	21 (42.86)	7 (43.75)	
CT guidance	130 (46.26)	93 (43.06)	28 (57.14)	9 (56.25)	

Abbreviations: PTA, percutaneous thermal ablation; IQR, interquartile range; ASA, American Society of Anesthesiologists; BMI, body mass index; HCC, hepatocellular carcinoma; NASH, non-alcoholic steatohepatitis; MR, magnetic resonance imaging, MELD, model for end-stage liver disease; AFP, alpha fetoprotein; ALBI, albumin–bilirubin; HCC, hepatocellular carcinoma; CT, computed tomography.

**Table 2:** Cumulative distant recurrence rate per year.

Cumulative Distant Recurrence Rate Per Year	Unifocal	Bifocal	Trifocal
6-month	10.7% (95% CI: 7.3–15.7)	38.8% (95% CI: 26.8–53.8)	50% (95% CI: 29–75.5)
1-year	21.8% (95% CI: 16.8–28.1%)	61.9% (95% CI: 48.5–75.4%)	68.8% (95% CI: 46.4–88.6%)
2-year	39.4% (95% CI: 32.6–47.1%)	72.9% (95% CI: 58.9–85.2%)	81.3% (95% CI: 59.8–95.4%)
3-year	51.1% (95% CI: 43–59.7%)	72.9% (95% CI: 58.9–85.2%)	81.3% (95% CI: 59.8–95.4%)

**Table 3:** Characteristics of the first distant recurrence.

Characteristics of the 1st Distant Recurrence	Unifocal	Bifocal	Trifocal	*p* Value
Tumor number recurrence ≤ 3	64/93 (68.8%)	29/37 (78.4%)	9/14 (64.3%)	0.5
Portal vein invasion or extra-hepatic metastasis	13/93 (14%)	5/37 (13.5%)	2/14 (14.3%)	0.82
Size of the largest nodule (mm)	14 (11–18)	14 (12–19)	13 (10–16)	0.59
Alpha-foetoprotein (ng/mL)	4.7 (3–10.3)	7.7 (4–31.9)	6.3 (4–13.1)	0.49
Non-Curative treatment *	42.4%	36.2%	38.5%	0.71

Numbers are ratio (percentages) or median (IQR). * Non-curative treatment was defined according BCLC [3] as trans-arterial chemoembolization, selective internal radiation therapy, systemic therapy or best supportive care.

**Table 4:** Univariate and multivariate Cox regression models to predict RFS (per patient.analysis).

	Univariate Analysis		Multivariate Analysis		Bootstrapping (200 Replications)	
Variables	Hazard Ratio (95% CI)	*p* Value	Hazard Ratio (95% CI)	*p* Value	Hazard Ratio (95% CI)	*p* Value
Patients						
Age	1.0 (0.99–1.02)	0.65				
Sex female vs. male	0.82 (0.58–1.17)	0.28				
Body Mass Index	0.99 (0.96–1.02)	0.36				
ASA (>2 vs. ≤2)	0.92 (0.68–1.23)	0.56				
Diabetes	0.90 (0.67–1.22)	0.52				
Metformin treatment	0.79 (0.51–1.22)	0.29				
Statin treatment	1.05 (0.71–1.56)	0.79				
Treatment-naïve patient	0.48 (0.36–0.66)	<0.001	0.46 (0.32–0.65)	<0.001	0.46 (0.31–0.67)	<0.001
Cirrhosis	1.23 (0.67–2.28)	0.50				
Child-Pugh (B vs. A)	1.63 (0.79–3.36)	0.19				
Cause of liver disease(vs. alcohol)						
Viral hepatitis or mixed	0.82 (0.56–1.18)	0.28				
Hemochromatosis and others	0.82 (0.38–1.76)	0.61				
NASH	0.52 (0.32–0.84)	0.007	0.67 (0.37–1.22)	0.190	0.67 (0.34–1.32)	0.245
Steatosis	1.09 (0.80–1.49)	0.59				
Laboratory Data						
AFP ≥ 100 vs. <100 ng/mL	3.27 (1.63–6.55)	0.001	3.31 (1.87–5.87)	<0.001	3.31 (1.70–6.45)	<0.001
AFP (per unit)	1.0 (1.0–1.0)	<0.001				
Prothrombin time	1.0 (0.98–1.01)	0.56				
Platelet count	1.0 (1.0–1.0)	0.81				
Albumin	0.97 (0.94–1.00)	0.057				
Bilirubin	1.02 (1.0–1.03)	0.11				
Creatinine	1.0 (1.0–1.0)	0.97				
MELD (>9 vs. ≤9)	1.17 (0.86–1.59)	0.33				
ALBI score 2 vs. 1	1.33 (0.97–1.82)	0.07				
HCC						
Bifocal HCC (vs. unifocal)	1.98 (1.27–3.06)	0.002	2.46 (1.60–3.77)	<0.001	2.46 (1.53–3.96)	<0.001
Trifocal HCC (vs. unifocal)	2.50 (1.19–5.23)	0.015	2.70 (1.16–6.29)	0.021	2.70 (1.02–7.08)	0.044
Tumor size < 2 vs. ≥2 cm	0.99 (0.73–1.35)	0.968				
Steatotic HCC	0.59 (0.39–0.88)	0.011	0.81 (0.52–1.26)	0.35	0.81 (0.51–1.28)	0.364
PTA						
PTA modality: MWA vs. RF	1.1 (0.81–1.48)	0.54				
PTA imaging guidance: US vs. CT	0.96 (0.71–1.30)	0.81				
Harrell’s C statistic: 0.69 AIC: 1256.97						

Abbreviations: CI, confidence interval; MWA, microwave ablation; RF, radiofrequency ablation; US, ultrasonography; CT, computed tomography; AIC, Akaike information criterion.

**Table 5:** Overall survival per year for each uni-, bi-, trifocal group.

Overall Survival Rate Per Year	Unifocal	Bifocal	Trifocal
1-year	98% (95%CI: 94.8–99.2%)	91.4% (95%CI: 78.7–96.7%)	100% (95 CI: not evaluable)
2-year	85.3% (95%CI: 78.9–89.9%)	81.1% (95%CI: 65.6–90.1%)	78.6% (95%CI: 47.3–92.5%)
3-year	77.9% (95%CI: 70.3–83.9%)	76.3% (95%CI: 58.1–87.5%)	27.5% (95%CI: 4.4–58.6%)

**Table 6:** Univariate and multivariate Cox regression models to predict OS (per patient Analysis).

Variables	Univariate Analysis		Multivariate Analysis		Bootstrapping (200 Replications)	
	Hazard Ratio (95% CI)	*p* Value	Hazard Ratio (95% CI)	*p* Value	Hazard Ratio (95% CI)	*p* Value
Patients						
Age	1.01 (0.99–1.04)	0.25				
Sex female vs. male	0.67 (0.35–1.29)	0.23				
Body Mass Index	1.02 (0.97–1.07)	0.48				
ASA (>2 vs. ≤2)	1.79 (1.10–2.95)	0.020	1.14 (0.76–2.78)	0.25	1.46 (0.67–3.18)	0.34
Diabetes	1.53 (0.94–2.48)	0.09				
Metformin treatment	1.0 (0.49–2.0)	1.0				
Statin treatment	1.44 (0.83–2.49)	0.190				
Treatment-naïve patient	0.59 (0.36–0.98)	0.041	0.42 (0.22–0.79)	0.007	0.42 (0.2–0.85)	0.017
Local recurrence	1.12 (0.65–1.93)	0.67				
Distant recurrence	1.50 (0.90–2.52)	0.12				
Non-Transplantable Recurrence	4.78 (2.66–8.58)	<0.001				
Cirrhosis	1.0 (0.46–2.1)	0.99				
Child-Pugh (B vs. A)	2.24 (0.47–10.62)	0.31				
Cause of liver disease(vs. alcohol)						
Viral hepatitis or mixed	0.85 (0.47–1.53)	0.59				
Hemochromatosis and others	0.24 (0.03–1.7)	0.15				
NASH	0.71 (0.33–1.52)	0.38				
Steatosis	1.08 (0.65–1.8)	0.76				
AFP ≥ 100 vs. <100 ng/mL	4.36 (2.12–8.96)	<0.001	3.03 (1.33–6.91)	0.008	3.03 (1.1–8.37)	0.032
AFP (per unit)	1.002 (1.001–1.003)	<0.001				
Prothrombin time	0.97 (0.96–0.99)	<0.001				
Albumin	0.93 (0.88–0.98)	0.006				
Bilirubin	1.04 (1.00–1.07)	0.026				
Creatinine	1.01 (1.00–1.01)	0.001				
MELD (>9 vs. ≤9)	2.28 (1.40–3.73)	0.001	2.84 (1.54–5.26)	0.001	2.84 (1.46–5.53)	0.002
ALBI score 2 vs. 1	1.48 (0.88–2.46)	0.14				
HCC						
Bifocal HCC (vs. unifocal)	1.1 (0.53–2.25)	0.80	1.60 (0.69–3.72)	0.27	1.60 (0.66–3.92)	0.30
Trifocal HCC (vs. unifocal)	2.75 (1.34–5.63)	0.006	3.30 (1.36–8.02)	0.008	3.31 (1.15–9.49)	0.026
Tumor size <2 cm (vs. ≥2 cm)	1.07 (0.64–1.79)	0.790				
Steatotic HCC	0.18 (0.056–0.56)	0.003	0.12 (0.01–0.89)	0.038	0.12 (3.2 ^−18–4.3^15)	0.912
PTA						
PTA modality: MWA vs. RF	1.34 (0.82–2.2)	0.24				
PTA imaging guidance US vs. CT	0.75 (0.48–1.24)	0.26				
Harrell’s C statistic: 0.79 AIC: 356.36						

## Data Availability

The data presented in this study are available on request from the corresponding author. The data are not publicly available because it was not mentioned in patient informed consent.

## Abbreviations

PTA	Percutaneous Thermal Ablation
HCC	Hepatocellular Carcinoma
BCLC	Barcelona Clinic Liver Cancer
US	Ultrasonography
RFS	Recurrence-Free Survival
AFP	Alpha-Fetoprotein
OS	Overall Survival
MELD	Model for End-Stage Liver Disease
NAFLD	Non-Alcoholic Fatty Liver Disease
ECOG	Eastern Cooperative Oncology Group
AASLD	American Association for the Study of Liver Disease
EASL	European Association for the Study of the Liver
BMI	Body Mass Index
ALBI	Albumin–Bilirubin
MRI	Magnetic Resonance Imaging
ASA	American Society of Anesthesiologists
CT	Computed Tomography
SIR	Society of Interventional Radiology
LTP	Local tumor progression
SD	Standard Deviation
IQR	Interquartile Range
AIC	Akaike Information Criterion
TACE	Transarterial Chemoembolization
OR	Odds Ratio
CI	Confidence Interval
HR	Hazard Ratio
NASH	Non-Alcoholic Steatohepatitis
